# *Ocrepeira klamt* sp. n. (Araneae: Araneidae), a novel spider species from an Andean páramo in Colombia

**DOI:** 10.1371/journal.pone.0237499

**Published:** 2020-08-24

**Authors:** Charlotte Hopfe, Bryan Ospina-Jara, Thomas Scheibel, Jimmy Cabra-García

**Affiliations:** 1 Department of Biomaterials, Universität Bayreuth, Bayreuth, Germany; 2 Department of Biology, Universidad del Valle, Cali, Valle del Cauca, Colombia; 3 Forschungszentrum für Bio-Makromoleküle, Universität Bayreuth, Bayreuth, Germany; 4 Bayreuther Zentrum für Kolloide und Grenzflächen, Universität Bayreuth, Bayreuth, Germany; 5 Bayreuther Materialzentrum, Universität Bayreuth, Bayreuth, Germany; 6 Bayreuther Zentrum für Molekulare Biowissenschaften, Universität Bayreuth, Bayreuth, Germany; 7 Bayrisches Polymerinstitut, Universität Bayreuth, Bayreuth, Germany; National Institute of Biology, SLOVENIA

## Abstract

Herein we describe *Ocrepeira klamt* sp. n. (Araneae: Araneidae), a new orb-weaving spider species from a Colombian páramo, which was formerly inaccessible for scientific studies due to decades long armed conflicts. Both, phenotypic and molecular data are used to confirm genus affiliation, and the new species is placed into phylogenetic context with other araneid spiders. Morphological characteristics and ecological notes of *Ocrepeira klamt* sp. n. are reported together with the sequence of the barcoding region of cytochrome c oxidase subunit I (COI) to provide a comprehensive description of the spider, facilitating future identification beyond taxonomic experts. With this study we contribute to the taxonomic knowledge that is required to inventory the hyper diverse yet threatened ecosystem of the Colombian páramos.

## Introduction

Biodiversity hotspots, priorities for conservation efforts due to their high number of endemic species, are thought to harbour most of undescribed organisms [1, 2]. Colombia is distinguished by accommodating two hotspots, Tumbes-Chocó-Magdalena and Tropical Andes, the latter being recognized for hosting the highest species richness and most endemics worldwide [1]. Yet, many areas remain unexplored due to a decades-long armed conflict, which has diminished only recently. Concerns are growing that these formally inaccessible territories are now more vulnerable to rapid human-induced change [3, 4], turning taxonomic inventories, monitoring and conservation initiatives into an urgent matter.

Located in the Tropical Andes are the Colombian páramos, neotropical alpine grassland ecosystems situated between the timberline and permanent snow fields. Characterized by swamps and wet grassland, conspicuous frailejones (*Espeletia*) and small shrub and forest patches, they are often referred to as ‘grassland isles within a sea of cloud forests’ [5–7]. This natural isolation and fragmentation has generated their high biodiversity and endemism, which, in combination with the páramos’ key role in Colombia’s hydrological system and growing disturbance through human activities, gives them particular significance and warrants special conservation efforts [6, 7].

Araneae (spiders) are a group of extremely diverse and abundant key predators, present in every terrestrial ecosystem [8–10]. They have been suggested as good ecological indicators, i.e. suitable to monitor functional changes in an ecosystem, as they respond readily to alterations in their biotic and abiotic environment [11]. Their utility depends on a profound knowledge on the identity of present species.

The family Araneidae Clerck, 1757 [12] is one of the most diverse spider families, currently including more than 3000 species in 178 genera [13]. Although Neotropical araneids are considered to be well-studied from a taxonomic perspective, as provided by H.W. Levi’s monographic revisions (see the complete list of Levi’s publications in [14]), several recent publications have revealed undescribed species across different genera [15–20]. In the quest to understand and protect biodiversity, uncovering species’ identities is a requisite, but it is a task that is rarely encouraged in our current times [21, 22]. Additionally, molecular and phenotypic data are seldom reported together, neglecting the potential of such a combined approach [23].

Here, we describe a new spider of the genus *Ocrepeira* Marx, 1883 [24] (Araneidae: Araneinae), and provide notes concerning its ecology from the Páramo Las Hermosas (Colombia), an area formerly inaccessible due to armed conflicts. To date, this orb-weaving genus includes 67 recognized species [13], which are found exclusively in the Americas and prevailingly inhabit high mountain environments, where the geographic isolation causes a high degree of endemism [25]. In providing the so far third ever sequence of the barcoding gene cytochrome c oxidase subunit I (COI) from the genus *Ocrepeira* [26, 27], and exploring the phylogenetic position of the new species, we complement the morphological description with molecular data.

## Materials & methods

### Study area & sampling

**Fig 1:**
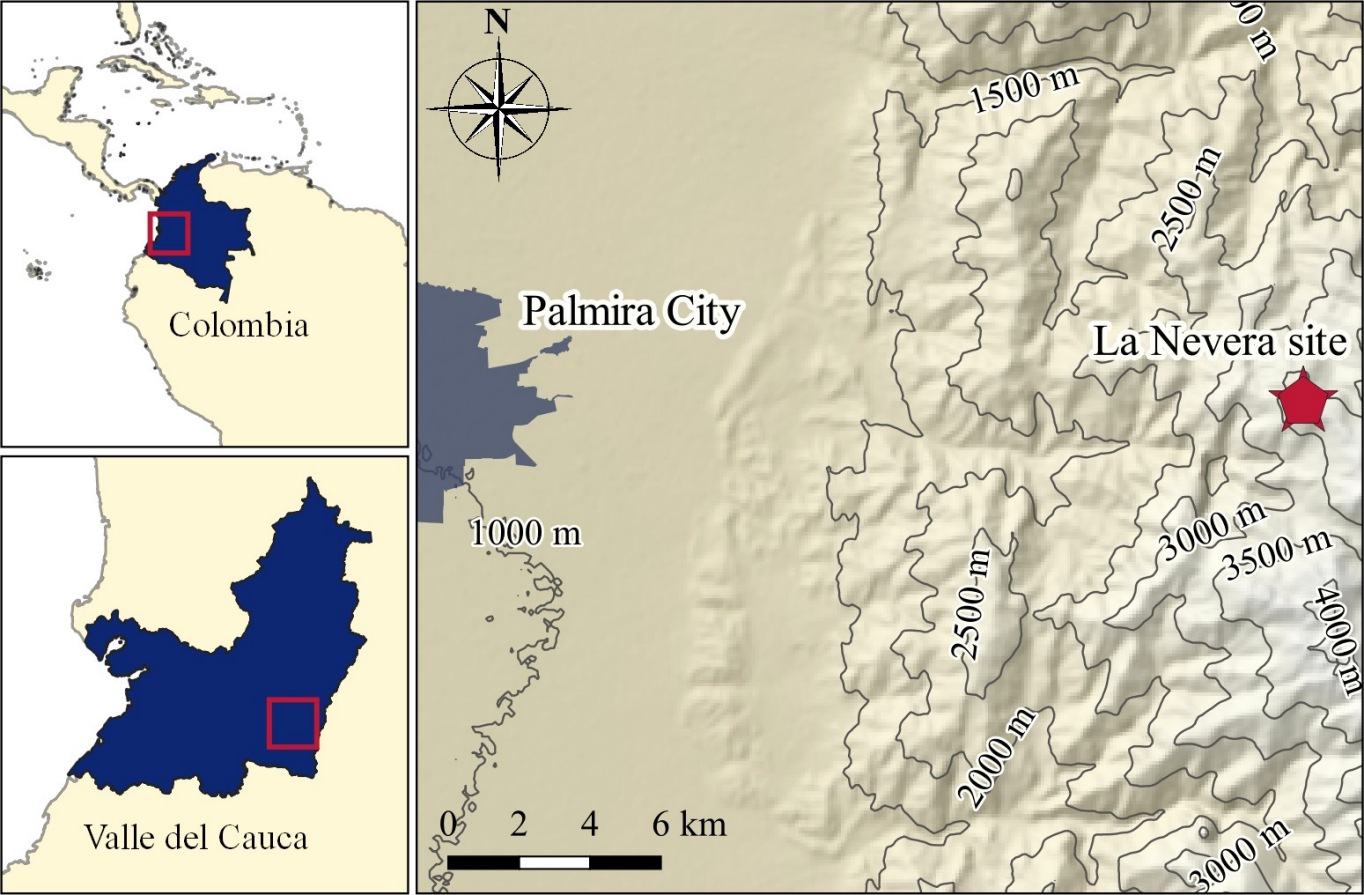
Sampling locality in Valle del Cauca (Colombia). This map is made with QGIS software v.3.10.4 based on the digital elevation model provided by the International Centre of Tropical Agriculture [32] and the data provided by the Departamento Administrativo Nacional de Estadística [33].

Spider sampling was conducted in an alpine grassland ecosystem in Valle del Cauca Colombia, the Páramo Las Hermosas, La Nevera locality (03°31'54.8'' N, 76°04'40.1'' W, elevation 3650 meters above sea level) in November of 2018 (Fig 1). Climatically, the area belongs to the montane, per-humid Holdridge life zone [28, 29], with a mean annual temperature of 7.4°C (calculated according to [30]) and mean annual total precipitations of 2400 mm (based on IMERG multi-satellite precipitation estimates [31] from the years 2014 to 2019). The study area is dominated by tussock grasses, dwarf shrubs and *Espeletia*, with few bush and tree patches (S1 Fig) [6]. We sampled the spiders through visual search and with a sweeping net, stored them individually in 95% alcohol and transferred them to a freezer (-20°C). The collection of specimens was performed under the permit #0526 granted by the Autoridad Nacional de Licencias Ambientales (ANLA) to the Universidad Icesi.

### Morphological analysis

Taxonomic description follows the format used by Cabra-García & Brescovit [34]. Specimens were examined using a Nikon C-PS stereomicroscope and a Nikon Eclipse Ci compound microscope. In order to visualize female internal genitalia, non-chitinous tissue was digested with pancreatin following the protocol described by Álvarez-Padilla & Hormiga [35]. We took the photos of preserved specimens and genitalia using a Nikon SMZ-1500 stereomicroscope equipped with a Nikon DS-Ri1 U3 camera and a Nikon Eclipse Ci compound microscope equipped with a Canon T5i camera of the in-house Laboratorio de Imágenes at Universidad del Valle (Cali, Colombia). Extended focal range images were composed using NIS-Elements Basic Research Software version 4.20.03. Morphological measurements were performed using the integrated ruler of a Nikon C-PS stereomicroscope with 13.4 x to 100 x magnification. All morphological measurements are in millimetres. Vouchers were deposited in the arachnological collection of the Museo de Entomología de la Universidad del Valle (MUSENUV) and the Instituto de Ciencias Naturales, Universidad Nacional de Colombia (ICN).

### Nomenclatural acts

The electronic edition of this article conforms to the requirements of the amended International Code of Zoological Nomenclature, and hence the new species name contained herein is available under that Code from the electronic edition of this article. This published work and the contained nomenclatural acts have been registered in ZooBank, the online registration system for the ICZN. The ZooBank LSIDs (Life Science Identifiers) can be resolved and the associated information viewed through any standard web browser by appending the LSID to the prefix "http://zoobank.org/". The LSID for this publication is: urn:lsid:zoobank.org:pub:5385566B-DCD5-4716-9B7F-5351690D3F4C. The electronic edition of this work was published in a journal with the eISSN: 1932-6203, has been archived and is available from the following digital repositories: PubMed Central, LOCKSS.

### Phylogenetic placement

Total DNA was extracted from one male and one female specimen of *O*. *klamt* sp. n. (MUSENUV-Ar 2092, DNA Vouchers A310 and A311). Thereby, for each specimen four legs were frozen with liquid nitrogen and ground up; the remainder of the spider was kept as a voucher. Subsequently, extractions including a negative control were carried out using the DNeasy Blood & Tissue Kit (Qiagen) including a RNAse (Promega) treatment (40 μg/ml, for 15 min at 37°C). DNA amplification of the COI locus was performed using existing invertebrate primers [36] adapted for the use with Araneomorphae spiders (HCO2198_spider TAWACYTCDGGRTGHCCAAAAAATCA; LCO1490_spider ATTCWACWAAYCAYAAGGATATTGG). Polymerase chain reactions (PCR) were performed in a 50 μl volume with 0.02 U/μl *Taq* DNA Polymerase, 1x ThermoPol buffer and 0.2 mM each dNTP (NEB), using 0.2 μM of each primer and between 30 and 50 ng of template DNA. A “touchdown” PCR profile was employed: Denaturation at 95°C for 30 s was followed by the first cycle set (6 repeats) with 20 s denaturation at 95°C, 60 s annealing at 48°C (-1°C per cycle) and 60 s extension at 68°C. The second cycle set (39 repeats) consisted of 20 s denaturation at 95°C, 60 s annealing at 43°C and 60 s extension at 68°C, followed by a final extension step of 5 min at 68°C. PCR products were purified using the Wizard® SV Gel and PCR Clean-Up System (Promega) and Sanger-sequenced (Eurofins Genomics). Contiguous sequences were assembled using the package consed/phred/phrap [37–40]. Once assembled, contigs were queried against the online NCBI BLAST database to check for possible contamination from external sources.

The taxon sampling for this study was guided by the most taxon-rich Araneidae phylogenetic analysis to date [26]. The sample includes all the members of the “Micrathenines” clade and representatives of Phonognathinae, Nephilinae, the “Caerostrines” clade and *Araneus necopinus* (Keyserling, 1887) [41]. *Zygiella x-notata* (Clerck, 1757) [12] (Araneidae: Phonognathinae) was used as the root. The higher Linnean ranks follow [42], for an alternative view see [43]. Phylogenetic relationships of the new species were inferred by concatenating the available nuclear 28S rRNA and mitochondrial COI sequences (Table 1). Similarity alignments *sensu* [44] were completed using MAFFT v.7.299b [45]. The COI gene was aligned using the L-INS-i method (command line: mafft—localpair—maxiterate 1000). After alignment, sequences were translated and checked for stop codons using Aliview v.1.18 [46]. The ribosomal gene was aligned using the E-INS-i method (command line: mafft—genafpair—maxiterate 1000) after [47].

**Table 1:** Taxon sampling and GenBank accession numbers.

Species	Cytochrome Oxidase I	28S
*Acacesia hamata* (Hentz, 1847) [55]	MK420048	MK425946
*Araneus necopinus* (Keyserling, 1887) [41]	MK420069	MK425961
*Gnolus cordiformis* (Nicolet, 1849) [56]	MK420112	MK426045
*Micrathena gracilis* (Walckenaer, 1805) [57]	MK420136	MK426013
*Micrathena militaris* (Fabricius, 1775) [58]		MK426014
*Micrathena sagittata* (Walckenaer, 1841) [59]	MK420137	MK426015
*Ocrepeira darlingtoni* (Bryant, 1945) [60]	MK227518	MK251221
*Ocrepeira darlingtoni* (Bryan, 1945) [60]	MK227519	
*Ocrepeira ectypa* (Walckenaer, 1841) [59]	MK420146	MK426020
*Ocrepeira klamt* sp. n.	MN991226	
*Ocrepeira klamt* sp. n.	MN991227	
*Scoloderus cordatus* (Taczanowski, 1879) [61]	MK420161	MK426035
*Trichonephila clavipes* (Linnaeus, 1767) [62]	MK420140	MK426047
*Verrucosa arenata* (Walckenaer, 1841) [59]	MK420168	MK426039
*Zygiella x-notata* (Clerck, 1757) [12]	MK420171	MK426042

The best tree was inferred in a maximum likelihood framework as implemented in IQTREE v.2.0 [48]. ModelFinder [49] was used to select the optimal partition scheme and substitution models (Table 2). Ten independent runs, including the calculation of the ultrafast Bootstrap [50] and the Shimodaira–Hasegawa approximate likelihood-ratio test (SH aLRT) [51], were conducted with the following command line: iqtree -s concat.nex -spp partition.nex.best_scheme.nex -B 1000 -alrt 1000 -pers 0.2 -nstop 1000.

**Table 2:** Best partition scheme and best-fit models selected by ModelFinder for the IQ-TREE analysis.

Subset	Partition names	Model
1	28S	GTR+F+R2
2	COI codon 1	GTR+F+I+G4
3	COI codon 2	TVM+F+I
4	COI codon 3	TN+F+R2

### Species boundaries

*Ocrepeira klamt* was foremost delimited from other species of the genus by comparing its morphological characters of the genitalia with the available literature [25, 52]. In addition, genetic distances were used to test for species boundaries within our *Ocrepeira* sample. The uncorrected intra- and inter-specific COI divergence among the available *Ocrepeira* species was calculated using MEGA X [53]. Species boundaries were tested using the Automatic Barcode Gap Discovery (ABGD) method [54]. ABGD analyses were carried out with the command-line version of the program, employing the simple distance metric (i.e. p-distance). The data were analysed using two different values for the parameters P_min_ (0.0001 and 0.001), P_max_ (0.1 and 0.2), and relative gap width (0.1 and 0.4), with the other parameters maintained at default values.

## Results

### Species description

*Ocrepeira klamt* Hopfe, Ospina-Jara, Scheibel & Cabra-García sp. n. urn:lsid:zoobank.org:act:B4AC6926-DAA8-488E80D3-B519BC6C497F

#### Types

Male holotype from Vereda La Nevera, Páramo Las Hermosas, Valle del Cauca, Colombia, 3°31'54.8'' N, 76°04'40.1'' W, elev. 3650 m, 11.XI.2018, C. Hopfe leg., deposited in MUSENUV-Ar 2090; Paratypes: 1♀ deposited in MUSENUV-Ar 2091; 8♀ 1♂ deposited in MUSENUV-Ar 2092, 1♂ deposited in ICN-Ar 12417, all the latter with the same data as the holotype.

#### Etymology

The specific epithet, a noun in apposition, honours the German teacher Ulrike Klamt.

#### Diagnosis

**Fig 2:**
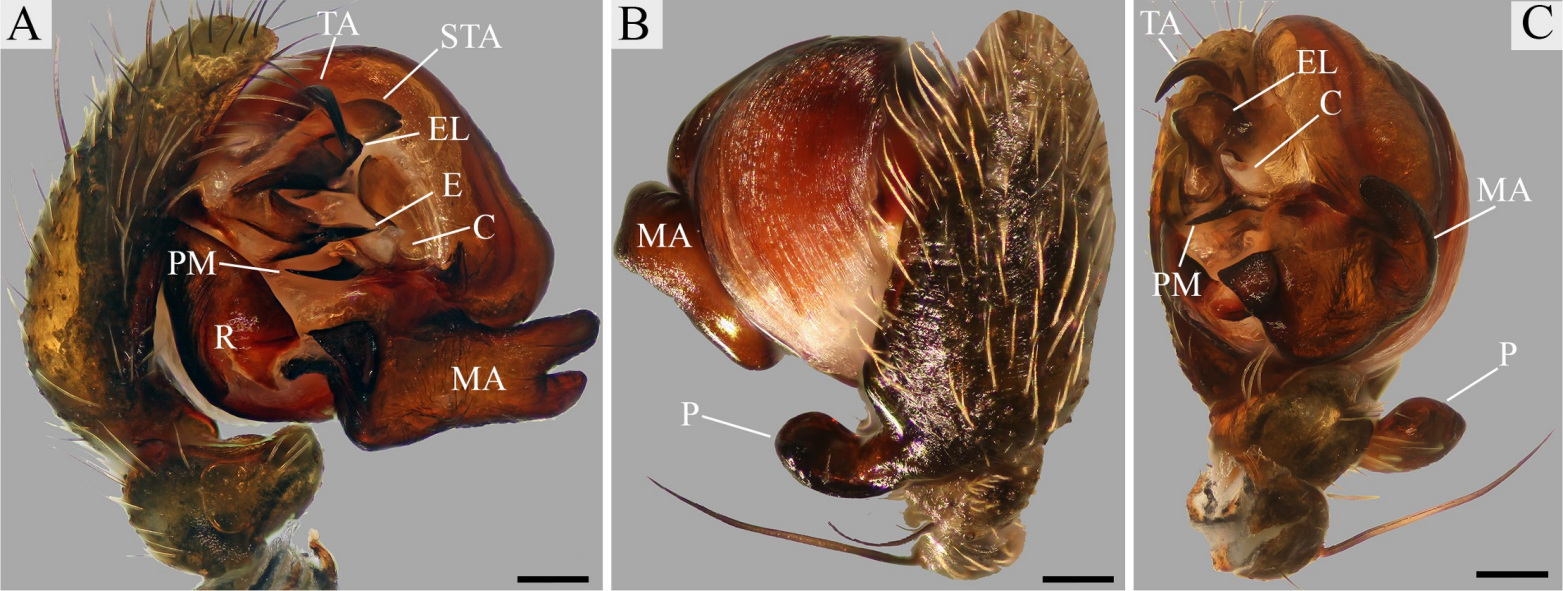
*Ocrepeira klamt* sp. n. holotype left palp (MUSENUV-Ar 2090). A: mesal; B: ectal; C: ventral. Scale bars: 100μm. Abbreviations: C, conductor; E, embolus; EL, embolus lamella; MA, median apophysis; P, paracymbium; PM, paramedian apophysis; R, radix; TA, terminal apophysis; STA, subterminal apophysis.

**Fig 3:**
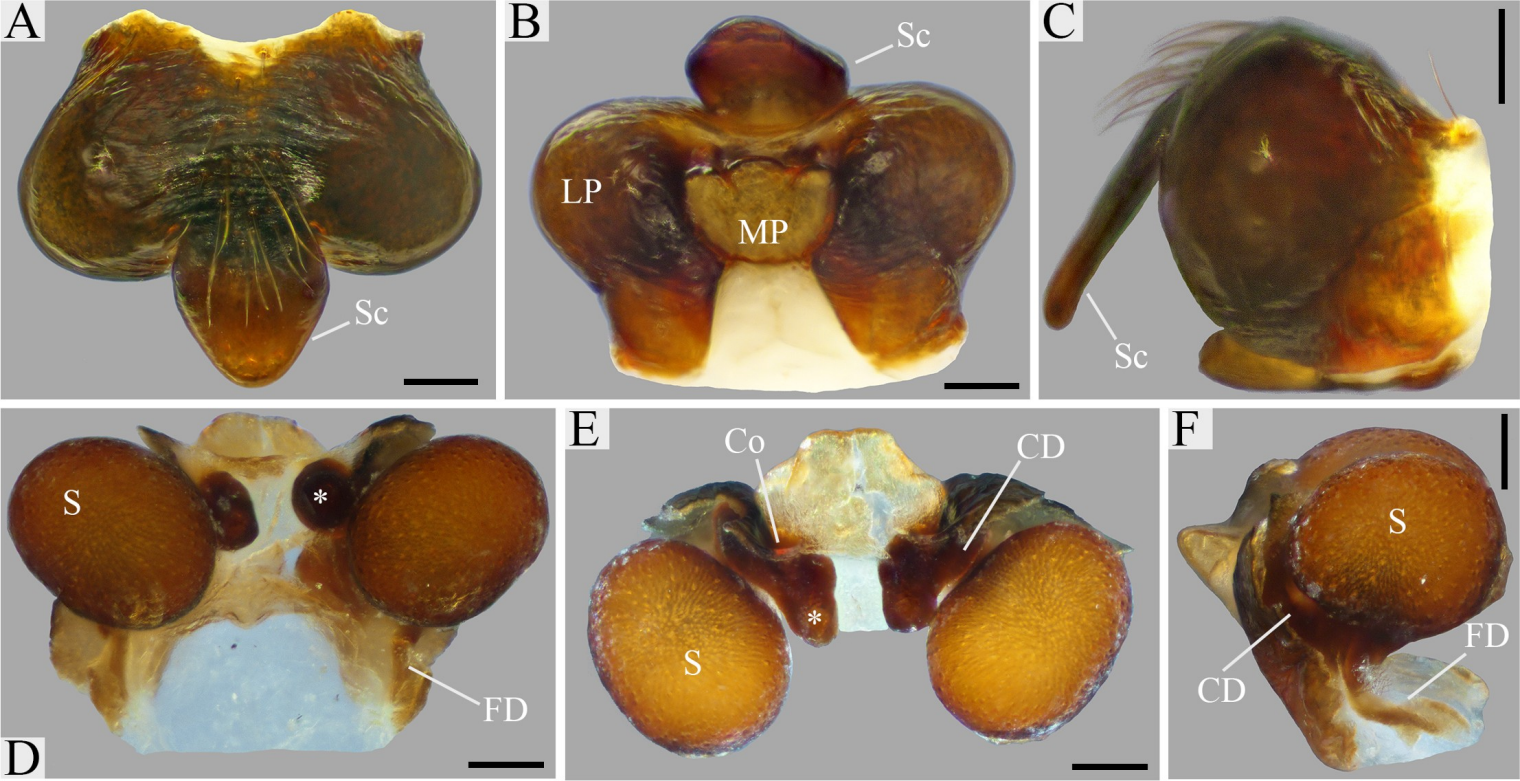
*Ocrepeira klamt* sp. n. A-C. Epigynum (MUSENUV-Ar 2092). A: ventral; B: posterior; C: lateral. D-F. Spermathecae and ducts (MUSENUV-Ar 2092). D: ventral, asterisk on CD basal enlargement; E: posteroventral, asterisk on CD basal enlargement. F: lateral. Scale bars: 100μm. Abbreviations: CD, copulatory duct; Co, copulatory opening; FD, fertilization duct; LP, lateral plate; MP, median plate; Sc, scape; S, spermathecae.

Males of *O*. *klamt* sp. n. resemble those of *O*. *valderramai* Levi, 1993 [25] by the presence of a triangular offset in the base of the median apophysis (Fig 2A and 2C). They are distinguished from the latter by the folded embolus lamella (Fig 2A) and the sharpened apical portion of the paramedian apophysis (Fig 2A and 2C). Females can be easily distinguished from all other *Ocrepeira* species by the three apical lobes of the median plate (Fig 3B).

#### Description

**Fig 4:**
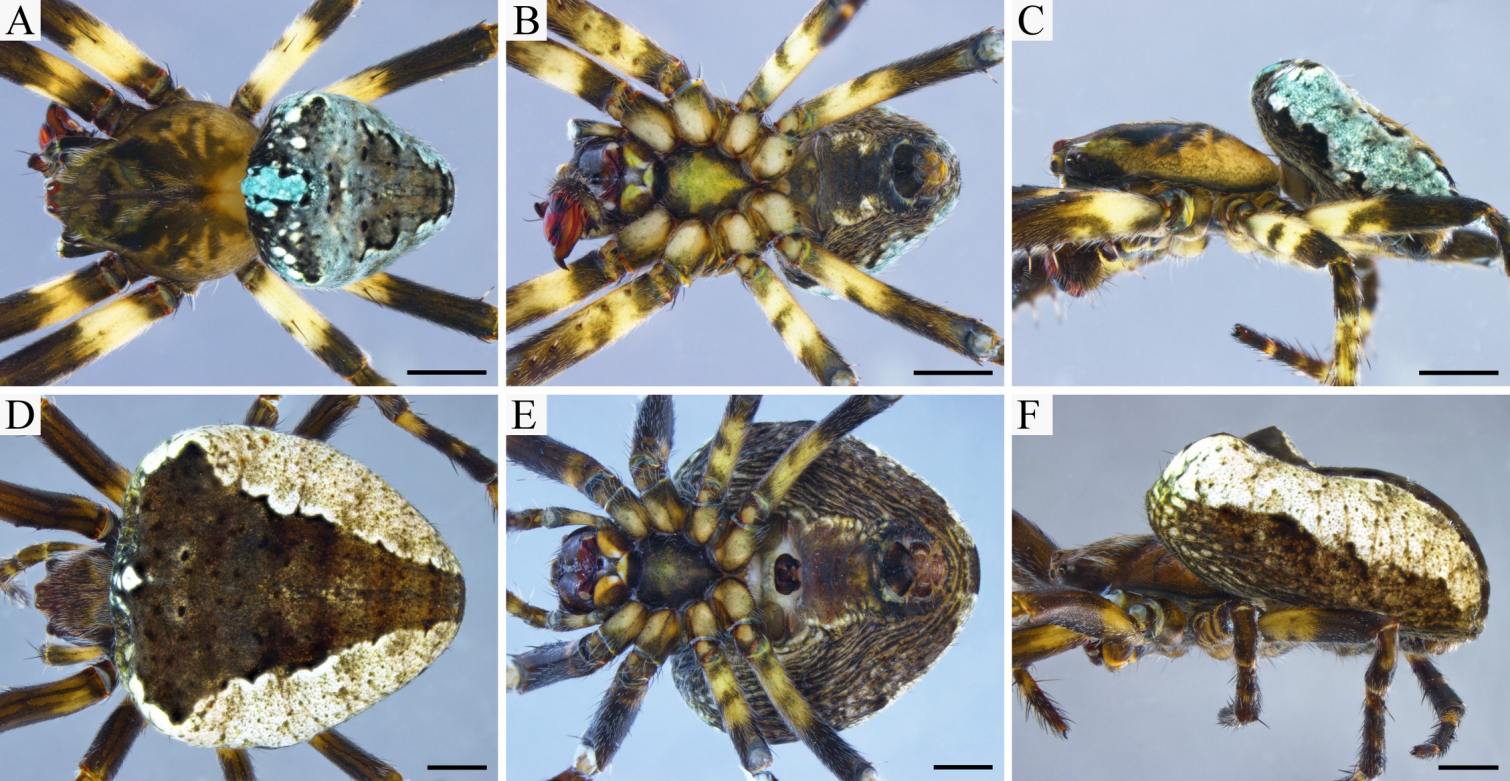
*Ocrepeira klamt* sp. n. habitus. A-C. Holotype (MUSENUV-Ar 2090). D-F. Paratype (MUSENUV-Ar 2091). A, D: dorsal view; B, E: ventral view; C, F: lateral view. Scale bars: 1 mm.

*Male (holotype)*. Fig 4A–4C. The carapace of the holotype is of khaki coloration, with a taupe brown cephalic region and dusky irregular areas in the thoracic region projecting to the lateral surface. Chelicerae black with the apical portion brown. Labium taupe brown with an anterior greenish rim. Endites walnut brown with greenish rims on their anterolateral margins. Sternum with a taupe brown fringe and a light greenish centre. Legs are taupe brown with buff brown and greenish patches, light coloured rings on the basal portion of femora, tibia, metatarsi and tarsi. The abdomen has two anterolateral and an anterior median hump; coloration of dorsum grey with a robin egg blue tint and a V-shaped beaver brown central pattern, delimited by black markings; anterior hump with Robin egg blue patch; venter with two white lines, black in the centre, sides taupe brown. Eight eyes in two transverse rows, recurved. Leg formula 1,2,4,3. For body measurements see Tables 3–5. Palp as in Fig 2A–2C. Median apophysis with two sub equal prongs (lower prong slightly shorter); without bulge on the lower edge; base with a triangular offset. Conductor with its basal portion sclerotized and with a membranous distal fold beneath the embolus. Paramedian apophysis spine-like, connected to the conductor. Radix without median outgrowth. Embolus pointed, with a basal sclerotized sharp projection. Embolus lamella folded apically. Subterminal apophysis rounded apically. Terminal apophysis spine-like.

**Table 3:** Eye diameters and distances for holotype and paratype of *Ocrepeira klamt* sp. n.

	Eye diameter (mm)	Eye distances (mm)			
Holotype ♂		AME	PME	ALE	PLE
AME	0.15	0.21	-	-	-
PME	0.13	0.14	0.17	-	-
ALE	0.11	0.28	0.38	1.02	-
PLE	0.11	0.46	0.50	0.06	1.22
Paratype ♀		AME	PME	ALE	PLE
AME	0.14	0.20	-	-	-
PME	0.15	0.15	0.21	-	-
ALE	0.12	0.26	0.39	1.17	-
PLE	0.13	0.46	0.54	0.07	1.33

AME: Anterior median eyes; PME: Posterior median eyes; ALE: Anterior lateral eyes; PLE: Posterior lateral eyes.

**Table 4:** Leg measurements of *Ocrepeira klamt* sp. n. holotype and paratype.

		Femur (mm)	Patella (mm)	Tibia (mm)	Metatarsus (mm)	Tarsus (mm)	Total (mm)
Holotype ♂	Leg I	3.55	1.32	2.85	2.60	1.12	11.44
	Leg II	2.75	1.17	2.15	2.38	1.00	9.45
	Leg III	1.90	0.84	1.15	1.15	0.69	5.73
	Leg IV	2.75	1.01	1.75	1.88	0.83	8.22
Paratype ♀	Leg I	3.30	1.55	2.65	2.65	1.23	11.38
	Leg II	3.10	1.55	2.45	2.65	1.15	10.90
	Leg III	1.98	0.92	1.30	1.23	0.75	6.18
	Leg IV	3.20	1.30	2.13	2.18	0.83	9.64

**Table 5:** Body measurements of *Ocrepeira klamt* sp. n. holotype and paratype.

		Holotype ♂	Paratype ♀
Abdomen length	(mm)	3.25	5.93
Abdomen width	(mm)	2.45	5.26
Carapace length	(mm)	2.90	3.05
Carapace width	(mm)	2.35	2.75
Carapace height	(mm)	0.75	0.95
Clypeus height	(mm)	0.17	0.17
Sternum length	(mm)	1.25	1.50
Sternum width	(mm)	1.08	1.43
Total length	(mm)	4.96	7.11

*Female (paratype)*. Fig 4D–4F. The female has a chocolate brown carapace, with similarly coloured chelicerae, labium and endites (the latter two having an anterior white rim). Sternum black. Legs chocolate to taupe brown, with lighter patches in the basal portion of femora, tibia, metatarsi and tarsi. The abdomen has two lateral humps and a small anterior median hump, and is longer than wide; the central part of the dorsum has a V-shaped chocolate brown pattern, the sides are dirty white and the anterior part of the abdomen shows a median white spot tinted grey-greenish; the venter is chocolate brown in the middle, bordered by two thin white lines, sides are taupe brown. Leg formula 1,2,4,3. For body measurements see Tables 3–5. Epigynum wider than long, sclerotized (Fig 3A–3C). Scape set off from base, longer than wide (Fig 3A and 3C). Median plate as wide as the maximum width of the lateral plates. The median plate with an undulated anterior rim with three lobes. Rounded spermathecae (Fig 3D–3F). Fertilization ducts shorter than copulatory ducts (Fig 3E and 3F). Copulatory ducts with a basal enlargement (Fig 3D and 3E).

#### Variation

**Fig 5:**
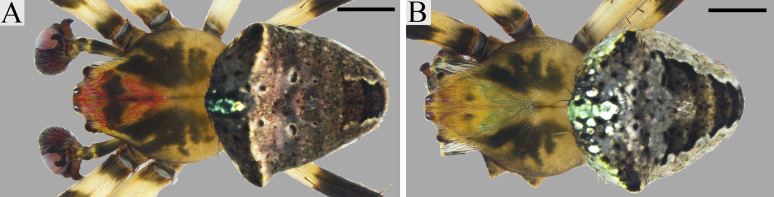
Colour variation of *Ocrepeira klamt* sp. n. males. Specimens preserved in alcohol. Scale bars: 1 mm.

*Male*. Coloration of paratypes lighter than in holotype, carapace dominated by shades of yellow. Tint of dorsum of abdomen can be green, and V-shaped pattern might be indistinct (Fig 5). White lines on the abdomen venter can be absent. Total length varies from 4.60 mm to 4.96 mm, carapace length from 2.75 mm to 3.18 mm (n = 3).

**Fig 6:**
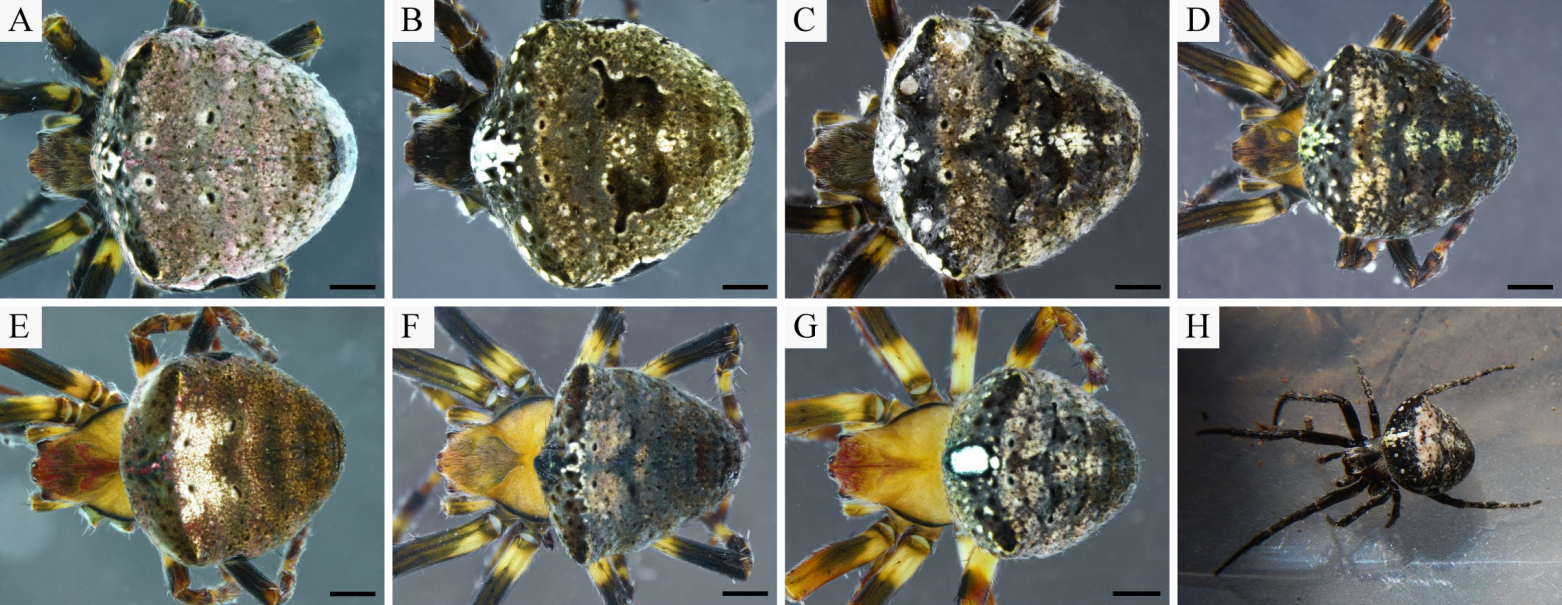
Colour variation of *Ocrepeira klamt* sp. n. females. A-G: variation of specimens preserved in alcohol; H: individual shortly after capture. Scale bars: 1 mm.

*Female*. Coloration of females varies from light (shades of yellows) to dark (shades of brown) (Fig 6). Seldom carapace with ruby red tint. Dorsum of abdomen very variable, colorations include: grey, walnut or taupe brown, ruby red, pink or green tint, grey-brown patterned. Venter with or without white lines or spots. Total length from 5.47 mm to 7.11 mm, carapace 2.85 mm to 3.55 mm (n = 9). On average females are 23% larger than males.

#### Distribution

Known only from the type locality.

#### Ecological notes

**Fig 7:**
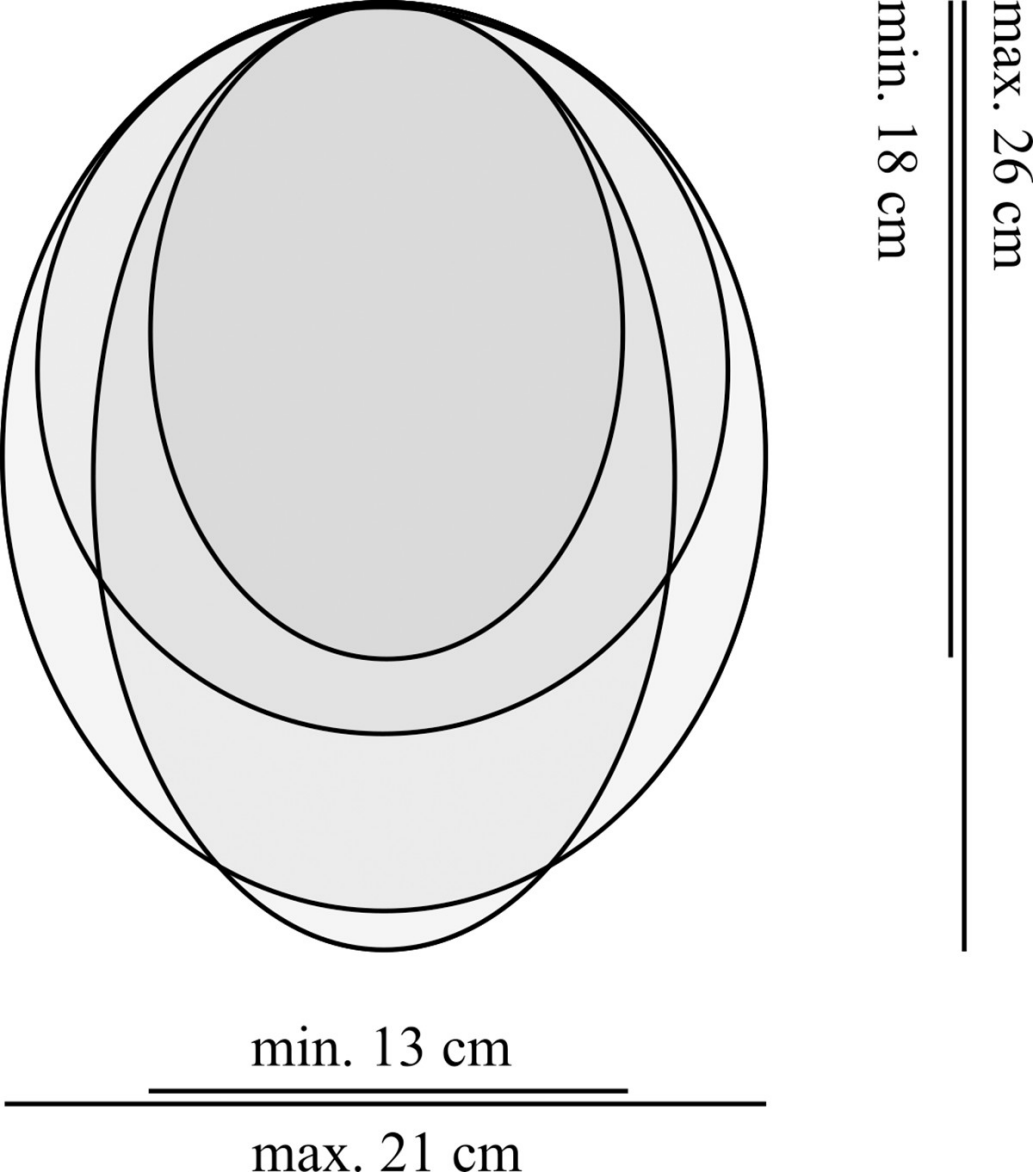
Representative web shapes of *Ocrepeira klamt* sp. n. Minimum and maximum measurements are given for both height and width of webs found in La Nevera.

As typical for *Ocrepeira*, *O*. *klamt* sp. n. builds orb webs. In shape they range from elliptical to round, and maximum heights and widths of around 26 cm x 21 cm, as well as minimum heights and widths of approximately 18 cm x 13 cm were observed in the field (Fig 7). They usually show a disorderly filled hub region. Webs were found in open, as well as slightly protected habitats (i.e. with nearby vegetation blocking e.g. strong winds) at heights between 0.4 m and 1.6 m above the ground. Spiders were spotted in their webs exclusively at night time, typically sitting in the hub, whereas during the day they could be collected sweeping the vegetation.

### Phylogenetic placement and species boundaries

COI sequences were generated for one male (voucher: A310, NCBI accession number: MN991226) and one female (voucher: A311, NCBI accession number: MN991227) *Ocrepeira klamt* sp. n. individual, with a length of 647 bp and 660 bp respectively.

**Fig 8:**
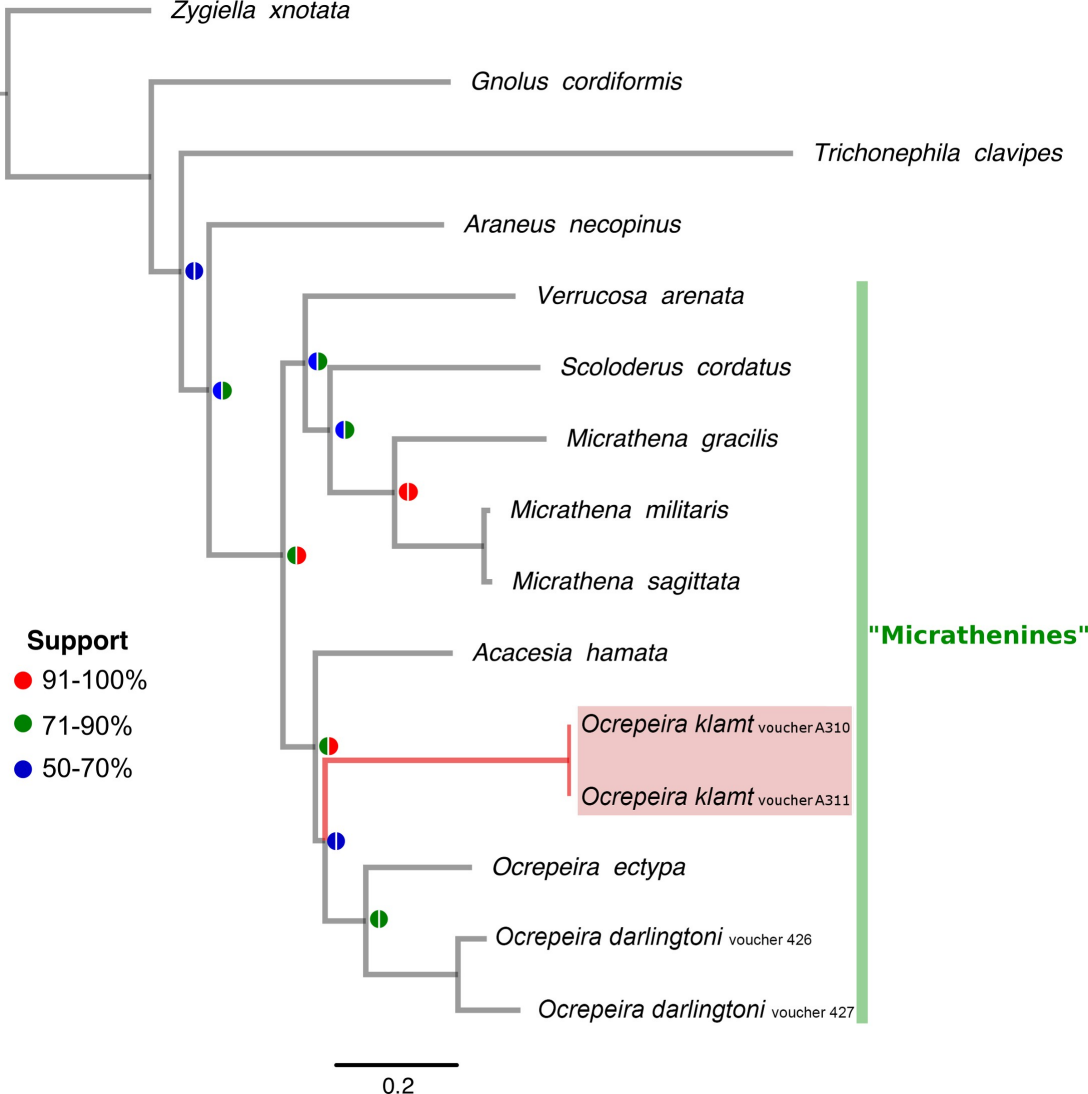
IQ-TREE optimal tree (log likelihood = -9629.087). Circles at nodes indicate the ultrafast bootstrap (left) and the Shimodaira–Hasegawa approximate likelihood-ratio test (right). Where applicable, voucher numbers are provided.

*Ocrepeira klamt* sp. n. is nested within the “Micrathenines” clade (Fig 8). *Ocrepeira klamt* sp. n. was recovered as the sister group of the clade *O*. *darlingtoni* (Bryant, 1945) [60] + *O*. *ectypa* (Walckenaer, 1841) [59], albeit with low support (Fig 8). The two specimens of *O*. *klamt* sp. n. yielded the same COI haplotype. The average intraspecific and interspecific distance among *Ocrepeira* species was 4.6% and 16.26%, respectively (Table 6). The ABGD analyses yielded three *Ocrepeira* species under each parameter setting (S1 Table).

**Table 6:** Percent uncorrected pairwise distances between COI sequences of *Ocrepeira* species.

Species & GenBank accession number	1	2	3	4	5
1 *Ocrepeira ectypa* (MK420146)	___				
2 *Ocrepeira darlingtoni* (MK227518)	15.6	___			
3 *Ocrepeira darlingtoni* (MK227519)	17.1	9.2	___		
4 *Ocrepeira klamt* (MN991226)	16.3	15.6	16.8	___	
5 *Ocrepeira klamt* (MN991227)	16.3	15.6	16.8	0	___

## Discussion

### Species identity and phylogenetic placement

#### Genus identification

The genus *Ocrepeira* was first mentioned by Marx [24], but it was Levi [25] who authored a comprehensive monograph for the genus, providing an updated diagnosis and a detailed morphological description. Levi [63] also proposed a key to the genera of araneids of the Americas, which allows to set apart *Ocrepeira* from similar genera considering female and male somatic and genital characters. Based thereupon we placed the new species in the genus *Ocrepeira* by confirming the presence of the following combination of characters in the examined specimens: carapace wide in the eye region, posterior median eyes facing dorsolaterally, clypeus height equal to one or at most two diameters of the anterior median eyes, abdomen with two anterior humps, pedicel attachment at the anterior half of the abdomen, and straight paramedian apophysis, all of them considered by Levi [25] as useful characters to differentiate *Ocrepeira* from putatively related genera such as *Acacesia* Simon, 1895 [64], *Wixia* O. Pickard-Cambridge, 1882 [65], *Wagneriana* O. Pickard-Cambridge, 1904 [66], *Parawixia* O. Pickard-Cambridge, 1904 [66] and *Pozonia* Schenkel, 1953 [67].

Additionally, we consider the phylogenetic placement in the DNA-based tree (Fig 8), where *O*. *klamt* sp. n. clusters together with *O*. *ectypa* and *O*. *darlingtoni*, albeit with low support. We acknowledge that due to the low support, our genus placement may be considered contentious. Nevertheless, an unequivocal placement would only be feasible after testing *Ocrepeira* monophyly using a comprehensive taxon sampling and molecular and morphological characters, a task beyond the scope of this paper.

#### Phylogeny

A recently published comprehensive phylogeny of araneid spiders, which was based on nuclear and mitochondrial genes, placed the species *Ocrepeira ectypa* as sister to *Acacesia hamata* (Hentz, 1847) [55] in the Micrathenines clade [26]. Similarly, the phylogenetic tree constructed from 28S and COI sequences recovers the Micrathenines clade and places *A*. *hamata* as the closest relative to *O*. *ectypa*, *O*. *darlingtoni* and *O*. *klamt* sp. n. with moderate (79% ultrafast bootstrap) to high (92% SH aLRT) node support (Fig 8). Other splits, however, differ between the two phylogenetic trees. Specifically, nodes that yielded ultrafast bootstrap values below 70% are inconsistent with results produced by [26]. A combined effect of few molecular markers and terminals in the present study may explain these inconsistencies.

#### Species delimitation

At species level, differences in genitalia structures are generally considered key for alpha-taxonomy due to their rapid evolutionary divergence [68]. When compared to the available scientific literature (i.e. [13, 25, 52]), both male and female *O*. *klamt* sp. n., exhibit a unique set of characters of the reproductive organs, setting them unequivocally apart from other *Ocrepeira* species (Figs 2 and 3). It is noteworthy that female internal genitalia across *Ocrepeira* species remain highly understudied. Nevertheless, a comparison of the available data (i.e. dissections of *O*. *darlingtoni* in [27]) with our cleared view (Fig 3D–3F) suggests that the morphology of the copulatory ducts could be highly informative to distinguish species.

The ABGD analyses among the available *Ocrepeira* sequences also suggested *O*. *klamt* sp. n. as a separate species. It is worth mentioning that while the low number of sequences per species available for the ABGD method may compromise its performance [54, 69, 70] have found that this method can yield similar results to other species delimitation tests, despite the low number of sequences per species.

#### Matching of the sexes and utility of COI

The matching of male and female individuals of an undescribed spider species is often problematic due to considerable sexual dimorphism. As one female *O*. *klamt* sp. n. individual was found in its web together with a male, a common species identity can however be inferred. Additional evidence comes from female and male COI sequences, which are identical (Table 6). COI, the so-called barcoding gene, is a useful tool to distinguish species, even those that are difficult to identify in most phyla with morphological taxonomic methods [71]. In the species-rich order Araneae, COI might be particularly helpful, making identification accessible to non-specialists and facilitating the identification of the more abundant juveniles, which cannot be distinguished phenotypically [72]. The utility of COI has been tested and confirmed for different spider families [73–75], and has been shown to yield an identification accuracy of 90% in Araneidae [75]. The here reported COI sequence from *O*. *klamt* sp. n. thus provides a valuable character to be used in conjunction with morphology for species identification. Being the third ever reported sequence from the genus *Ocrepeira* [26, 27], it adds to the growing reference database of COI sequences for spiders [72, 76].

### Ecological notes

*Ocrepeira klamt* sp. n. is a typical representative of the genus, producing vertical orb webs with a filled hub region (e.g. similar to *O*. *saladito* Levi, 1993 [25]) and being nocturnal [25]. Although other species of this genus, like *O*. *jamora* Levi, 1993 [25], *O*. *valderramai*, *O*. *cuy* Levi, 1993 [25], *O*. *abiseo* Levi, 1993 [25] and *O*. *tinajillas* Levi, 1993 [25], can likewise be found at high altitudes, the specimens collected at an elevation of 3650 m make *Ocrepeira klamt* sp. n. the highest recorded species of its genus (compare to [25, 52, 77]). So far, *O*. *klamt* sp. n. is solely known from the type locality, and the island characteristic of páramo ecosystems suggests that the species has a small distribution range, similarly to that of other known *Ocrepeira* species [6, 25].

## Conclusion

The discovery of *Ocrepeira klamt* sp. n. contributes to a steady stream of new floral and faunal descriptions from the Colombian páramos [78–80] that is increasing since the diminution of the armed conflict. Due to extremely high speciation rates even in comparison with other ultra-diverse ecosystems of the Tropical Andes, the páramos can be considered a ‘hotspot within a hotspot’ [81], making them a number one priority for monitoring and conservation efforts. With the description of a novel araneid species from the Páramo Las Hermosas, a region with previously unexplored spider fauna due to its inaccessibility caused by armed conflict, we contribute to the taxonomic knowledge required to inventory, monitor and ultimately protect this important ecosystem. Thereby, the combination of molecular with morphological data facilitates the accurate association between male and female individuals, provides two independent sources of support for genus affiliation and expands the utility of the data.

## Supporting information

S1 Fig Vegetation in Páramo Las Hermosas at La Nevera locality. (TIF)

S1 Table Results of the Automatic Barcode Gap Discovery (ABGD) analyses. (DOCX)
