# Supplementary figures and images for: Ocrepeira klamt sp. n. (Araneae: Araneidae), a novel spider species from an Andean páramo in Colombia
Source: PLoS One. 2020 Aug 24;15(8):e0237499. doi: 10.1371/journal.pone.0237499 (PMC7446859; doi:10.1371/journal.pone.0237499)

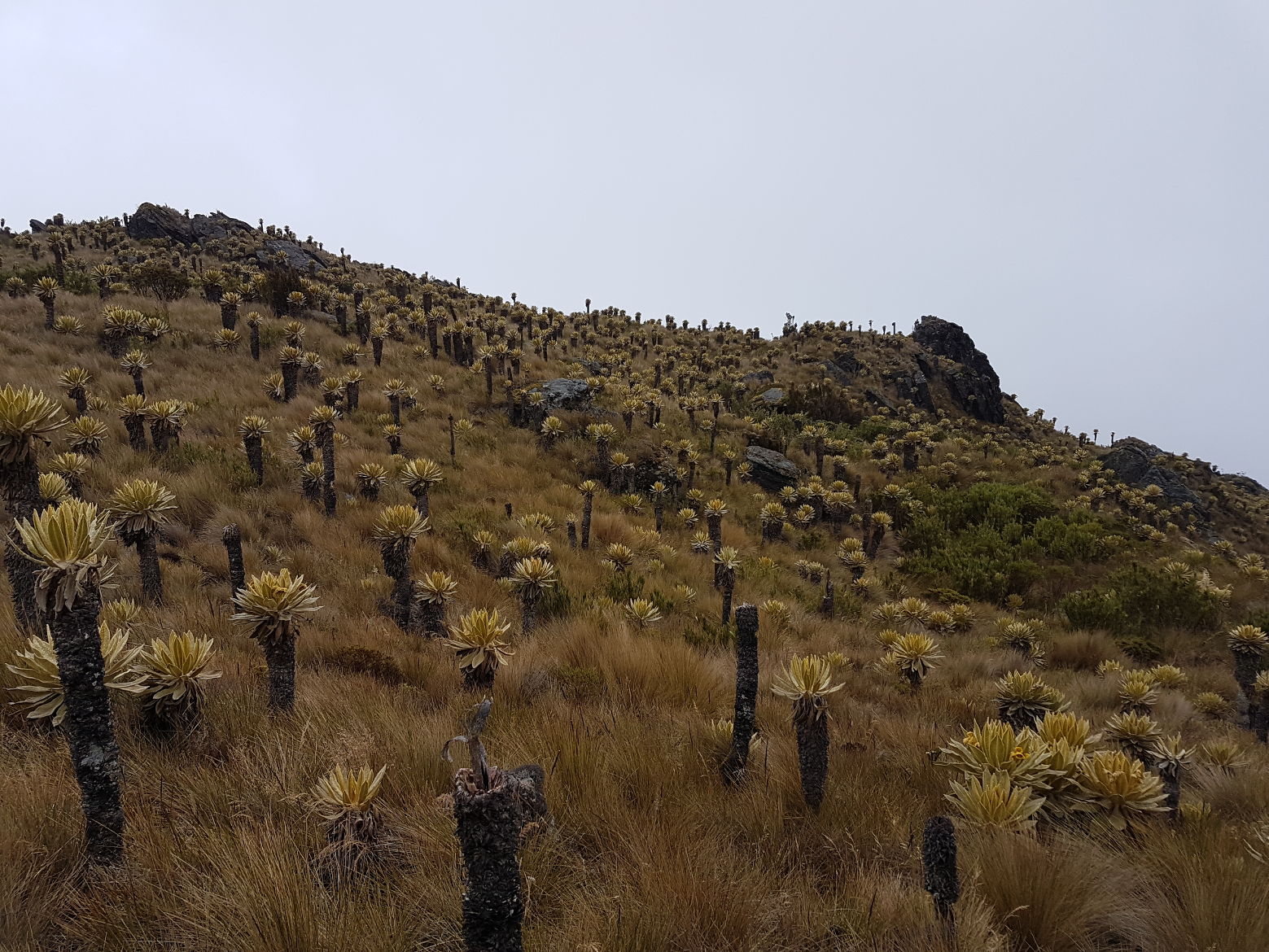

Supplement: S1 Fig — (TIF) [file pone.0237499.s001.tif]
