# Supplementary material for: Ocrepeira klamt sp. n. (Araneae: Araneidae), a novel spider species from an Andean páramo in Colombia
Source: PLoS One. 2020 Aug 24;15(8):e0237499. doi: 10.1371/journal.pone.0237499 (PMC7446859; doi:10.1371/journal.pone.0237499)
Supplement: S1 Table — (DOCX) [file pone.0237499.s002.docx]

**S1 Table: Results of the Automatic Barcode Gap Discovery (ABGD) analyses.**

| P_min_/P_max_ | X | Partition | Prior intraspecific divergence (P) | | | | | | | | |
| --- | --- | --- | --- | --- | --- | --- | --- | --- | --- | --- | --- |
|  |  |  | 0.0001 | 0.0002 | 0.0005 | 0.0013 | 0.0029 | 0.0068 | 0.0159 | 0.0369 | 0.0860 |
| 0.0001/0.2 | 0.1 | Initial | 3 | 3 | 3 | 3 | 3 | 3 | 3 | 3 | 3 |
|  |  | Recursive | 3 | 3 | 3 | 3 | 3 | 3 | 3 | 3 | 3 |
| 0.0001/0.2 | 0.4 | Initial | 3 | 3 | 3 | 3 | 3 | 3 | 3 | 3 | 3 |
|  |  | Recursive | 3 | 3 | 3 | 3 | 3 | 3 | 3 | 3 | 3 |
|  |  |  | 0.0010 | 0.0017 | 0.0028 | 0.0046 | 0.0077 | 0.0129 | 0.0215 | 0.0359 | 0.0599 |
| 0.001/0.1 | 0.1 | Initial | 3 | 3 | 3 | 3 | 3 | 3 | 3 | 3 | 3 |
|  |  | Recursive | 3 | 3 | 3 | 3 | 3 | 3 | 3 | 3 | 3 |
| 0.001/0.1 | 0.4 | Initial | 3 | 3 | 3 | 3 | 3 | 3 | 3 | 3 | 3 |
|  |  | Recursive | 3 | 3 | 3 | 3 | 3 | 3 | 3 | 3 | 3 |
